# Supplementary material for: L-selectin mechanochemistry restricts neutrophil priming in vivo
Source: Nat Commun. 2017 May 12;8:15196. doi: 10.1038/ncomms15196 (PMC5437312; doi:10.1038/ncomms15196)
Supplement: Supplementary Information — Supplementary Figures and Supplementary Tables [file ncomms15196-s1.pdf]

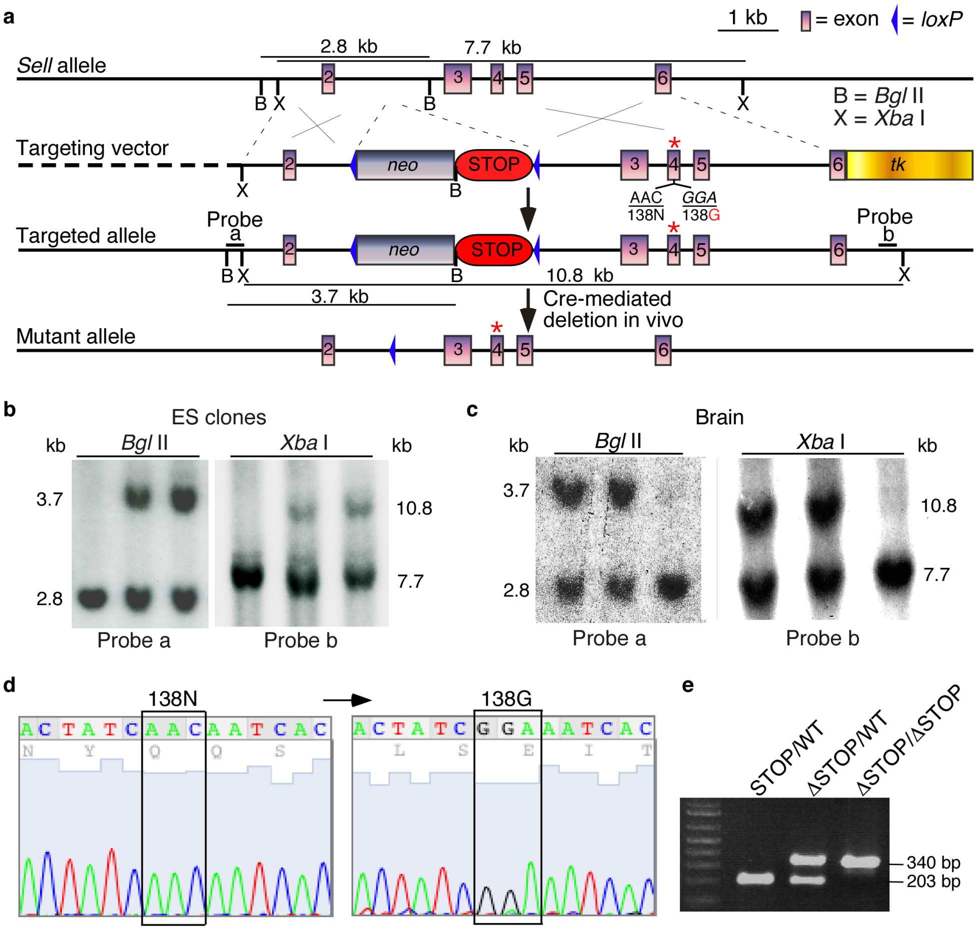

Supplementary Figure 1. Generation of knockin mice expressing L-selectinN138G. (**a**) Schematics of the *Sellg* allele (top), the targeting vector, the targeted allele in ES cells, and the mutant allele in mice. The targeting vector contained 5'- and 3'- *Sell* homologous sequences, a floxed *neostop* cassette inserted in intron 2, a mutated exon 4, and a thymidine kinase gene (*tk*). The targeted allele lost *tk* after homologous recombination in ES cells. The mutant allele was obtained by breeding mice expressing the targeted allele with mice expressing Cre recombinase at the 1-cell zygote stage, leaving one *loxP* site after deletion of the *neostop* cassette. Asterisks indicate the point mutations in exon 4. Probes and restriction fragment lengths for Southern blot analysis are indicated. (**b** and **c**) Southern blot analysis of targeted ES clones and mouse brains. (**d**) Sequencing of blood leukocyte cDNA confirmed nucleotide changes and corresponding single amino acid substitution from asparagine (N) to glycine (G) at position 138 of L-selectin (boxed regions). (**e**) PCR of genomic DNA from murine tails to confirm deletion of *neostop* cassette. PCR bands are 203 bp for WT allele and 340 bp for mutant allele after deletion of *neostop* cassette (ΔSTOP).

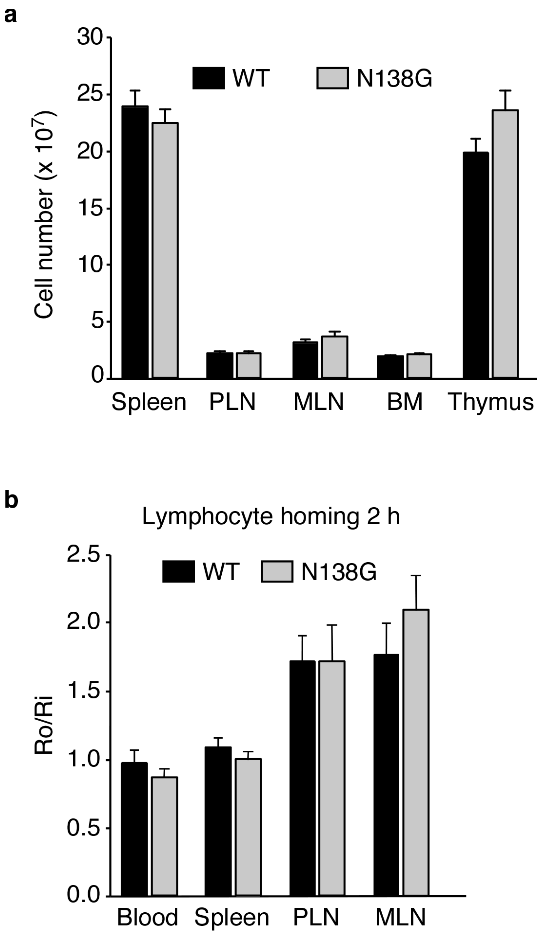

Supplementary Figure 2. Normal organ cellularity and lymphocyte homing in N138G mice.

(**a**) Number of cells isolated from spleen, peripheral lymph node (PLN, inguinal and cervical), mesenteric lymph node (MLN), bone marrow (BM, one femur and one tibia), and thymus of 12-week old mice of both genders. Data are mean ± s.e.m. from 11 mice per group. (**b**) Homing of circulating lymphocytes to organs. 10^7 CMFDA-labeled splenocytes from WT or N138G mice mixed with 10^7 CMTMR-labeled WT splenocytes were injected intravenously into WT mice. An aliquot of mixtures was used to assess the input ratio (Ri) calculated as CMFDA-positive cells per CMTMR-positive cells. After 2 h, the number of homed donor cells from different lymphoid organs was identified by flow cytometry. The resident ratio (Ro) of CMFDA-positive cells per CMTMR-positive cells in each organ was calculated. Results are expressed as the homing index Ro/Ri. Data are mean ± s.e.m. of 3 experiments.

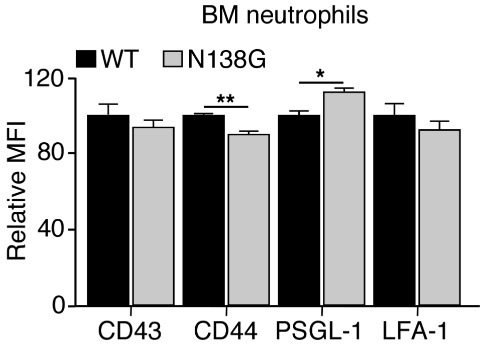

Supplementary Figure 3. Surface expression of glycoproteins on BM neutrophils from WT and N138G mice.
Mean fluorescence intensity (MFI) of the indicated glycoprotein is depicted. Data are mean ± s.e.m. of 5 mice per group. *, $P < 0.05$; **, $P < 0.01$ (two-tailed Student's *t*-test).

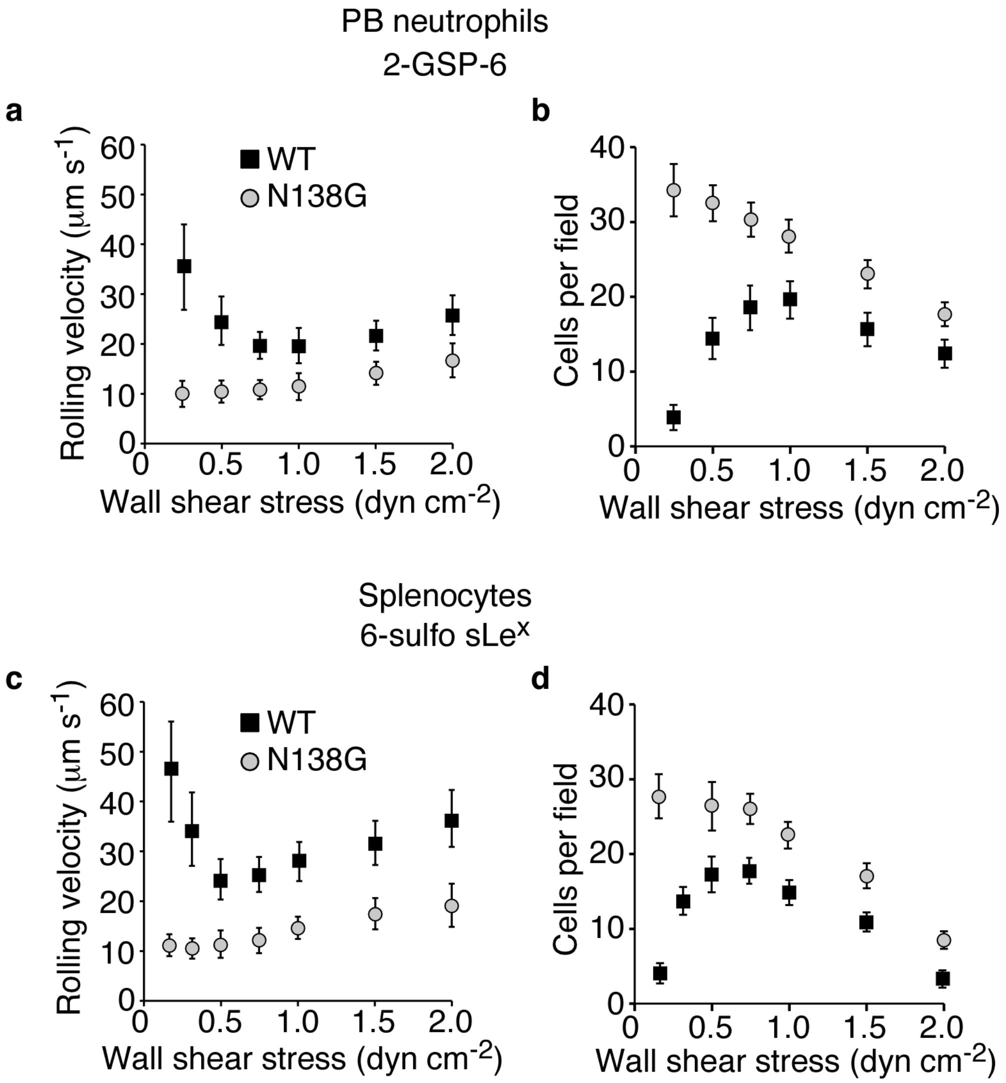

Supplementary Figure 4. Altered L-selectin mechanochemistry in N138G mice reduces the shear threshold for rolling of PB neutrophils and splenocytes on L-selectin ligands.

Flow chamber assays of L-selectin-dependent interactions of PB neutrophils with 2-GSP-6 (surrogate for PSGL-1) (**a**, **b**) or of splenocytes with 6-sulfo-sLe^x (surrogate for PNAd) (**c**, **d**). Mean rolling velocities (**a**, **c**) and rolling cell numbers (**b**, **d**) are shown. Data are mean ± s.d. from 5 experiments.

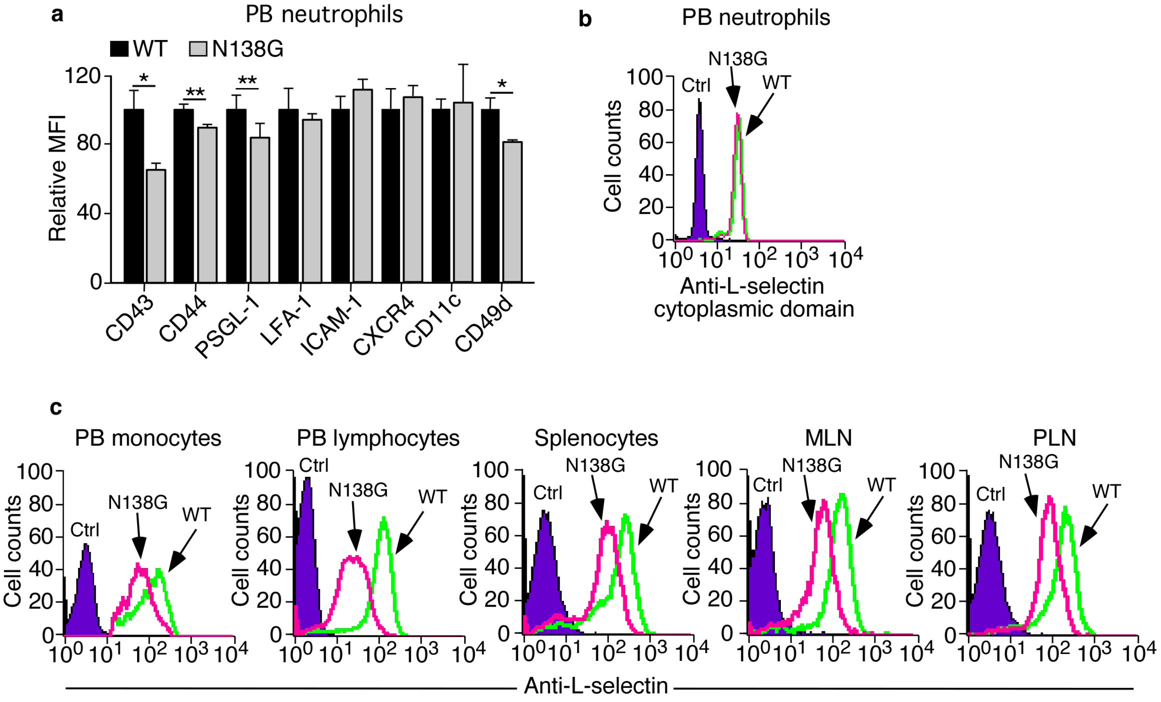

Supplementary Figure 5. Surface expression of glycoproteins on leukocyte subsets from WT and N138G mice.

(**a**) Mean fluorescence intensity (MFI) of the indicated glycoprotein on PB neutrophils. Data are mean ± s.e.m. of 5 mice per group. (**b**) Histogram of permeabilized PB neutrophils stained with polyclonal antibody to the cytoplasmic domain of L-selectin. (**c**) Histograms of the indicated cell population stained with anti-L-selectin mAb. MLN, lymphocytes from mesenteric lymph node; PLN, lymphocytes from peripheral lymph node. Histograms are representative of 3 mice per group. *, $P < 0.05$; **, $P < 0.01$ (two-tailed Student's *t*-test).

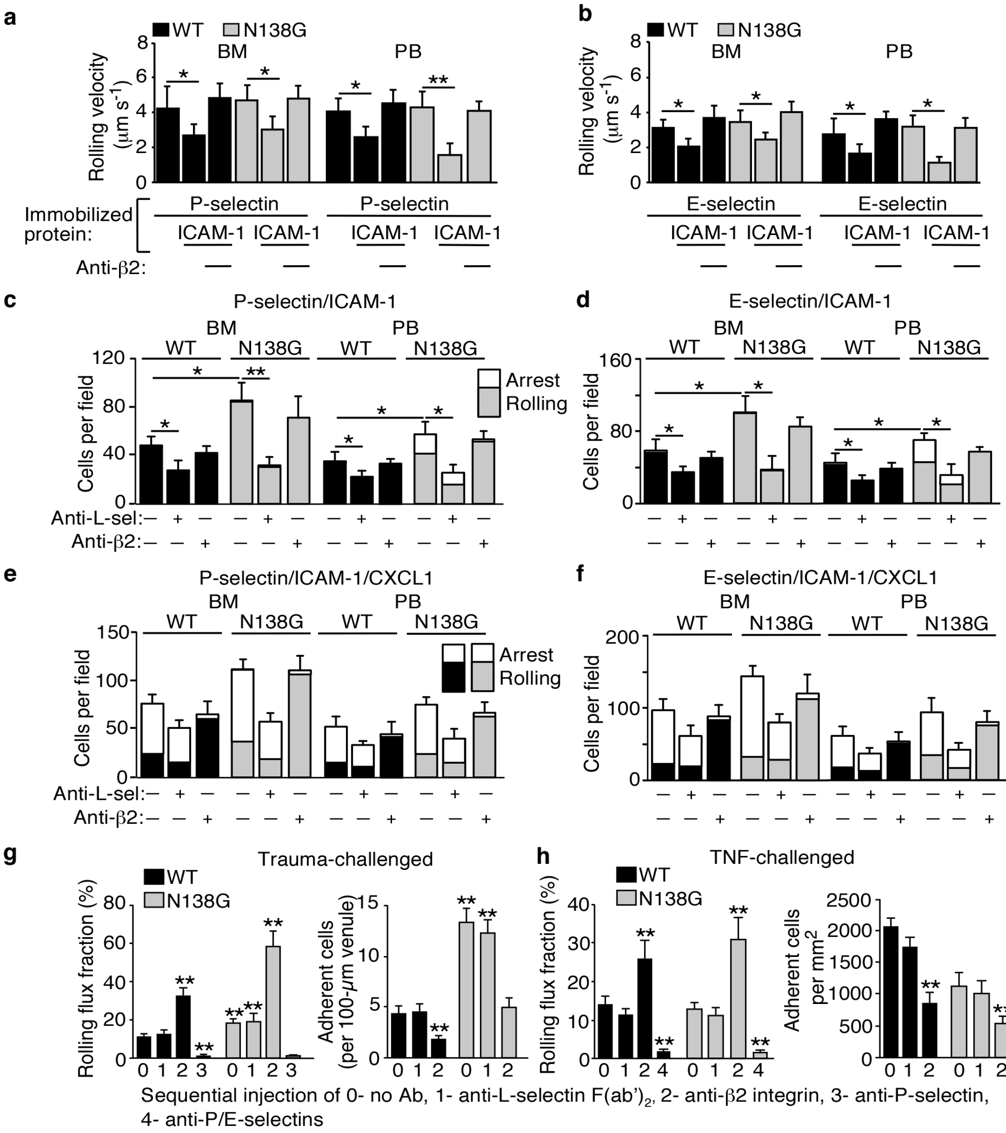

Supplementary Figure 6. Neutrophil priming in N138G mice has minimal effect on β2 integrin-dependent adhesion in vitro or in vivo.

(**a**-**d**) Rolling velocities and numbers of neutrophils rolling on P- or E-selectin with or without coimmobilized ICAM-1 in the presence or absence of anti-β2 integrin mAb or anti-L-selectin mAb. (**e**, **f**) Numbers of neutrophils rolling and arrested on P- or E-selectin coimmobilized with ICAM-1 and CXCL1 in the presence or absence of anti-β2 integrin mAb or anti-L-selectin mAb. The wall shear stress in **a**-**f** was 1 dyn cm^{-2}. Data are mean ± s.d. from 5 experiments, with 5 mice in each experimental group. (**g**, **h**) Leukocyte adhesion in venules of cremaster muscle after challenge with trauma or TNF. The rolling flux fraction and number of adherent leukocytes were measured before and after sequential injection of blocking mAbs as indicated. Data are mean ± s.e.m. from 5 experiments. *, $P < 0.05$; **, $P < 0.01$ (two-tailed Student's *t*-test).

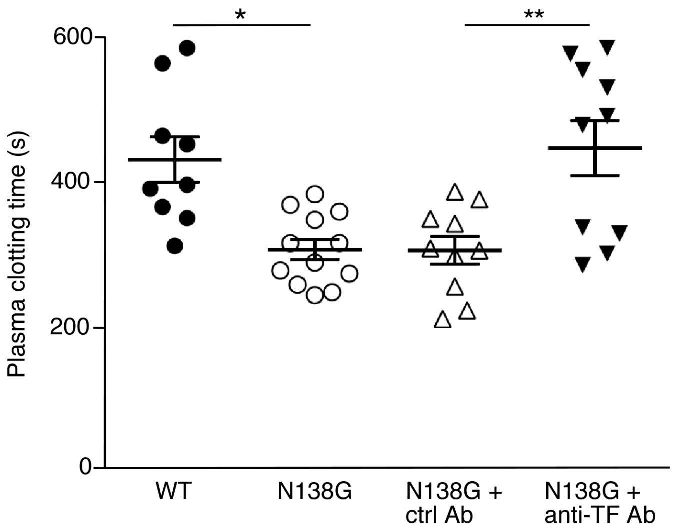

Supplementary Figure 7. Plasma clotting times are shorter in N138G mice.
Plasma was re-calcified by adding an equal volume of 25 mM $CaCl_2$ and incubated at 37°C with or without control antibody or blocking anti-tissue factor antibody. The time to clot was measured in a coagulometer. Symbols represent individual mice. Horizontal bars indicate the mean ± s.e.m. from 4 experiments. *, $P < 0.05$; **, $P < 0.01$ (two-tailed Student's *t*-test).

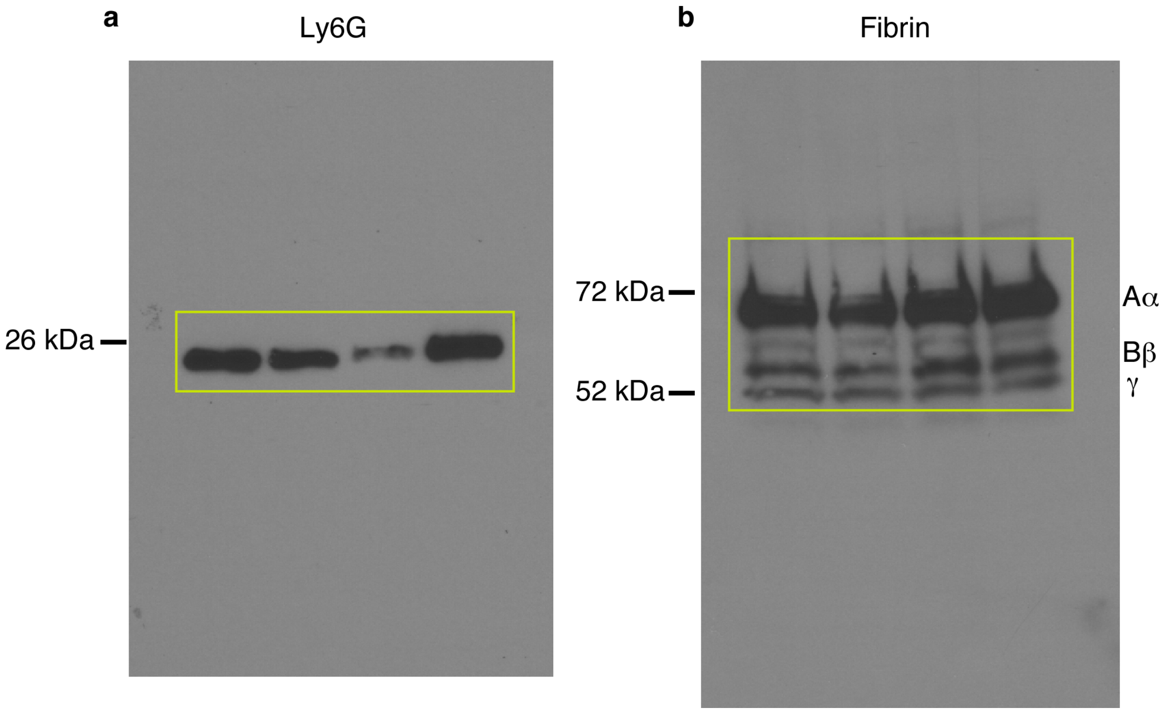

Supplementary Figure 8. Full Western blots for Fig. 7g.
Western blots of thrombus lysates were probed with antibodies to Ly6G (**a**) and fibrin (**b**). Areas within the yellow boxes are shown in Fig. 7g.

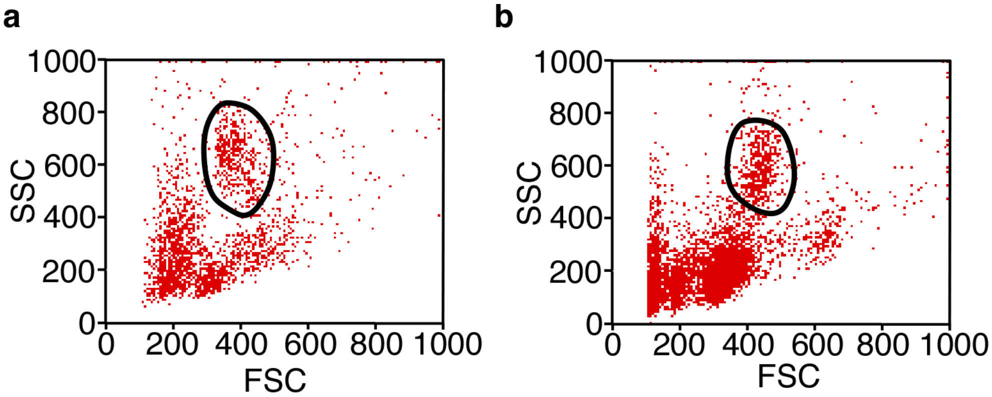

Supplementary Figure 9. Gating strategy for flow cytometry.
The gating strategy is shown to define (**a**) BM neutrophils for Fig. 1b, or (**b**) PB neutrophils for Fig. 3a and Fig. 4a, b, d, e, and f.

Supplementary Table 1

Peripheral blood cell counts

	WT (n = 25)	N138G (n = 21)
Total leukocytes	2.9 ± 0.2	2.7 ± 0.2
Neutrophils	0.6 ± 0.04	0.8 ± 0.1
Lymphocytes	2.2 ± 0.2	1.8 ± 0.1*
Monocytes	0.10 ± 0.01	0.11 ± 0.01
Platelets	1204 ± 55	1273 ± 61
Erythrocytes	9.3 ± 0.1	8.8 ± 0.1**

Data are mean ± s.e.m. x 10^3 μl^{-1} (leukocytes and platelets) or x 10^6 μl^{-1} (erythrocytes).
*, $P < 0.05$; **, $P < 0.01$ versus WT (two-tailed Student's *t*-test).

Supplementary Table 2

Plasma cytokine/chemokine levels (mean ± s.e.m., pg ml^{-1})

Cytokine/chemokine	WT (n = 10)	N138G (n = 9)	*P* value
IL-1α	15 ± 1	14 ± 2	0.48
IL-1β	339 ± 34	390 ± 2	0.26
IL-2	ND	ND	
IL-3	27 ± 4	26 ± 3	0.86
IL-4	ND	ND	
IL-5	13 ± 1	16 ± 1	0.16
IL-6	15 ± 2	18 ± 2	0.32
IL-9	ND	ND	
IL-10	41 ± 8	118 ± 23	0.01
IL-12 (p40)	321 ± 15	340 ± 24	0.51
IL-12 (p70)	387 ± 45	371 ± 47	0.81
IL-13	456 ± 55	491 ± 40	0.62
IL-17	47 ± 6	38 ± 3	0.21
Eotaxin	1555 ± 170	1724 ± 164	0.50
G-CSF	71 ± 5	77 ± 6	0.49
GM-CSF	102 ± 10	102 ± 12	0.99
IFN-g	65 ± 6	66 ± 5	0.90
CXCL1	45 ± 4	42 ± 4	0.56
MCP-1	302 ± 27	314 ± 24	0.76
MIP-1α	24 ± 3	25 ± 2	0.93
MIP-1β	69 ± 7	78 ± 8	0.43
RANTES	16 ± 2	12 ± 2	0.24
TNF	1160 ± 162	1255 ± 124	0.65

ND, not detectable. *P* value determined by two-tailed Student's *t*-test.
